# Leukotriene B4 Levels in Human Atherosclerotic Plaques and Abdominal Aortic Aneurysms

**DOI:** 10.1371/journal.pone.0086522

**Published:** 2014-01-27

**Authors:** Pleunie van den Borne, Sander W. van der Laan, Sandra M. Bovens, Dave Koole, Mark C. Kowala, Laura F. Michael, Arjan H. Schoneveld, Sander M. van de Weg, Evelyn Velema, Jean-Paul de Vries, Gert J. de Borst, Frans L. Moll, Dominique P. V. de Kleijn, Paul H. A. Quax, Imo E. Hoefer, Gerard Pasterkamp

**Affiliations:** 1 Laboratory of Experimental Cardiology, University Medical Center Utrecht, Utrecht, The Netherlands; 2 Interuniversity Cardiology Institute of the Netherlands, Utrecht, The Netherlands; 3 Department of Vascular Surgery, University Medical Center Utrecht, Utrecht, The Netherlands; 4 Lilly Research Laboratories, Eli Lilly and Company, Indianapolis, Indiana, United States of America; 5 Department of Vascular Surgery, Antonius Hospital Nieuwegein, Nieuwegein, The Netherlands; 6 Cardiovascular Research Institute and Surgery, National University Hospital Singapore, Singapore, Singapore; 7 Department of Surgery, Leiden University Medical Center, Leiden, The Netherlands; 8 Einthoven Laboratory for Experimental Vascular Medicine, Leiden University Medical Center, Leiden, The Netherlands; Brigham and Women’s Hospital, Harvard Medical School, United States of America

## Abstract

**Background:**

Leukotriene B4 (LTB4) has been associated with the initiation and progression of atherosclerosis and abdominal aortic aneurysm (AAA) formation. However, associations of LTB4 levels with tissue characteristics and adverse clinical outcome of advanced atherosclerosis and AAA are scarcely studied. We hypothesized that LTB4 levels are associated with a vulnerable plaque phenotype and adverse clinical outcome. Furthermore, that LTB4 levels are associated with inflammatory AAA and adverse clinical outcome.

**Methods:**

Atherosclerotic plaques and AAA specimens were selected from two independent databases for LTB4 measurements. Plaques were isolated during carotid endarterectomy from asymptomatic (n = 58) or symptomatic (n = 317) patients, classified prior to surgery. LTB4 levels were measured without prior lipid extraction and levels were corrected for protein content. LTB4 levels were related to plaque phenotype, baseline patient characteristics and clinical outcome within three years following surgery. Seven non-diseased mammary artery specimens served as controls. AAA specimens were isolated during open repair, classified as elective (n = 189), symptomatic (n = 29) or ruptured (n = 23). LTB4 levels were measured similar to the plaque measurements and were related to tissue characteristics, baseline patient characteristics and clinical outcome. Twenty-six non-diseased aortic specimens served as controls.

**Results:**

LTB4 levels corrected for protein content were not significantly associated with histological characteristics specific for vulnerable plaques or inflammatory AAA as well as clinical presentation. Moreover, it could not predict secondary manifestations independently investigated in both databases. However, LTB4 levels were significantly lower in controls compared to plaque (p = 0.025) or AAA (p = 0.017).

**Conclusions:**

LTB4 levels were not associated with a vulnerable plaque phenotype or inflammatory AAA or clinical presentation. This study does not provide supportive evidence for a role of LTB4 in atherosclerotic plaque destabilization or AAA expansion. However, these data should be interpreted with care, since LTB4 measurements were performed without prior lipid extractions.

## Introduction

Atherosclerosis is a progressive inflammatory disease occurring in the middle and larger arteries that can result in gradual luminal narrowing or acute thrombotic occlusion due to atherosclerotic plaque rupture [1]. During initiation of atherosclerosis, the activated endothelium expresses adhesion molecules resulting in adhesion and infiltration of leukocytes [2], [3]. Monocyte recruitment in the vascular wall can be mediated by different cytokines, such as leukotrienes.

Leukotriene B4 (LTB4), an end product of the arachidonate 5-lipoxygenase (ALOX5) pathway of the arachidonic acid (AA) pathway, is a potent chemoattractant and pro-inflammatory lipid mediator derived from membrane phospholipids. Upstream of ALOX5, phospholipases A2, like protein kinase C (PKC), are involved in translocation of ALOX5 to the nuclear envelope. After translocation, LTB4 can be generated from ALOX5 on the nuclear membrane of inflammatory cells, predominantly granulocytes, macrophages, mast cells, but also vascular smooth muscle cells (SMCs) endothelial cells and platelets [4], [5]. The known receptors for LTB4 are B leukotriene receptor 1 (BLT1) and BLT2. BLT1 is known as the high affinity receptor for LTB4 and is primarily, but not exclusively, expressed by inflammatory cells. [6], [7]. LTB4 exerts broad inflammatory actions. After binding to BLT1, it affects leukocytes by stimulating the formation of the CD34 positive bone marrow cells and their subsequent migration into the blood circulation. LTB4 also enhances cell motility and endothelial adhesion molecule expression, promoting leukocyte transmigration into the tissue. Furthermore, LTB4 enhances cell survival and leukocyte activation [8]. Components of the ALOX5 pathway and its downstream LTA4 hydrolase (LTA4H), lead to local production of different leukotrienes in the atherosclerotic plaque [9]. Although different components of the leukotriene pathway have been suggested to play a role in atherogenesis and plaque instability [10], the specific role of LTB4 is still unclear. The functional relevance of LTB4 and its receptors in atherosclerotic disease have been investigated in mouse models deficient for ApoE or LDLR. Based on these results, specific antagonists for ALOX5 pathway components have been developed to inhibit lesion development.

The ALOX5 pathway, and LTB4 specifically, not only plays a role in atherogenesis, also aneurysm formation has been linked to this pathway. A mouse model for abdominal aortic aneurysm (AAA) formation revealed that BLT1 deficiency results in a lower incidence of AAA with a reduced tissue inflammation [11]. The aforementioned results suggest that leukotrienes, and LTB4 in particular, are actively involved in plaque instability and aneurysm formation [12]. Although *ex vivo* production of LTB4 has been associated with in human atherosclerotic plaques in the past [13], evidence in human studies supporting the role of LTB4 in both advanced atherosclerosis and AAA in secondary clinical outcome is scarce.

We investigated the expression of LTB4 in advanced human atherosclerotic plaques and AAA specimens separately in two independent databases. We related expression levels to atherosclerotic plaque and AAA characteristics in a side-by-side analysis. Furthermore, we studied the possible link between LTB4 levels and clinical characteristics of patients suffering from atherosclerosis or AAA, separately.

## Materials and Methods

### Ethics Statement

All patients scheduled for CEA or open AAA repair in the two participating centers (St. Antonius Hospital Nieuwegein and University Medical Center Utrecht) are asked to participate in the corresponding studies. The Medical Ethics Committee of the two centers approved the studies and all patients provided a written informed consent.

### Biobanks

The Athero-Express (AE) biobank and the Aneurysm-Express biobank are two independent ongoing multi-center biobanks with a longitudinal study design. The AE biobank comprises carotid atherosclerotic plaque specimens obtained from patients that underwent carotid endarterectomy (CEA) and the Aneurysm-Express biobank comprises AAA specimens obtained from patients undergoing open AAA surgical repair. The biomaterials collected during the procedures are processed in the laboratory according to a standardized protocol described previously [14], [15]. After surgery, the patients undergo a 3-year clinical follow-up. For the present study, we included 375 atherosclerotic specimens from patients included in the AE biobank between May 2002 and October 2009 and 241 AAA specimens from patients included in the Aneurysm-Express biobank between April 2003 and April 2009.

### Patient Populations

#### Athero-Express biobank

Patients included for CEA were classified as asymptomatic (no clinical symptoms related to the carotid luminal stenosis >70%) (n = 58) or symptomatic (n = 317) prior to surgery. The symptoms can be categorized into minor clinical symptoms (i.e. transient ischemic attack, amaurosis fugax or retinal infarction; n = 228) or major clinical symptoms (i.e. stroke; n = 89).

#### Aneurysm-Express biobank

The indications for intervention following the diagnosis of AAA were based on current guidelines and included: AAA diameter exceeding 55 mm for males and between 50 and 55 mm for females, rapidly expanding aortic diameters (≥5 mm in 6 months with a minimum of 40 mm), saccular aneurysms, and symptoms attributable to the AAA or rupture of the AAA. Patients selected for open surgical repair were classified as elective (n = 189), ruptured (n = 23), or symptomatic AAA (n = 29) prior to surgery.

### Tissue Examination

#### Immunohistochemistry (CEA)

According to a standardized protocol, the plaque was divided into equal segments of 5 mm in length along the longitudinal axis. The segment with the greatest plaque burden was considered as the culprit lesion and subjected to (immuno)histochemical examination. Plaque segments were fixed in formalin (4%) and embedded in paraffin. Consecutive sections were stained with Hematoxylin & Eosin (H&E) for general overview including calcifications, and intraplaque hemorrhages, Elastic van Gieson (EvG) for elastin content, Picro Sirius Red (PSR) for collagen content, α-actin for smooth muscle cells (SMC), CD68 for macrophages, CD66b for neutrophils and CD34 for microvessel density. Macrophage and SMC content were measured quantitatively by computerized analysis using AnalySIS 3.2 software (Soft Imaging Systems GmbH, Münster, Germany) and reported as percentage positive staining per plaque area [14]. Microvessel density in the plaque was quantified in three hotspots and expressed as an average number of vessels per hotspot [16]. Neutrophil content was quantified as total number of CD66b positive cells in the plaque [17].

Presence of collagen, calcification and intraplaque hemorrhage was scored semi-quantitatively as no, minor, moderate or heavy staining in different locations in the plaque. Intraplaque hemorrhage was defined as a hemorrhage in the plaque without any signs of cap rupture and/or thrombus formation. Size of lipid core was visually estimated as a percentage of total plaque area using H&E and PSR staining. A plaque containing >40% lipid core was considered as atheromatous. Previous histological examinations reported good intra and inter observer reproducibility for the quantitative and semi-quantitative measurements [16].

A random selection of plaques was stained for the LTB4 receptor, BTL1, (n = 19) and for ALOX5 (n = 28). Sections were pretreated by boiling in citrate buffer as antigen retrieval solution (20 minutes, pH 6.0). BLT1 was detected by incubation with a polyclonal rabbit anti-human BLT1 antibody (1 hour at RT, 1∶800, Cayman Chemicals LtD, Michigan, USA). ALOX5 was detected using polyclonal rabbit anti-human ALOX5 antibody (1 hour at RT, 1∶100, Sigma Aldrich, St. Louis, USA). For both stainings, Brightvision poly HRP-anti-rabbit IgG (30 minutes at RT, ready to use, Immunologic, Duiven, The Netherlands) was used as a secondary antibody. The signal was visualized using diaminobenzidine (DAB) (DAKO, Carpinteria, USA). Sections were counterstained with haematoxylin.

#### Immunohistochemistry (AAA)

According to a standardized protocol, biopsies of the ventral wall of the AAA at the site of maximal diameter were collected during open repair and dissected into 5 mm segments. The middle segment was formalin fixed (4%) and embedded in paraffin. Tissue was analyzed using (immuno)histochemistry. Sections were stained for H&E, EvG, PSR, vascular SMCs, and CD68. In addition, T-lymphocytes (CD3), B-lymphocytes (CD20) and plasma cells (CD138) were identified. Analysis was performed as described previously [15].

#### Validation of LTB4 measurements after tissue isolation from AAA specimens

**Figure 1:**
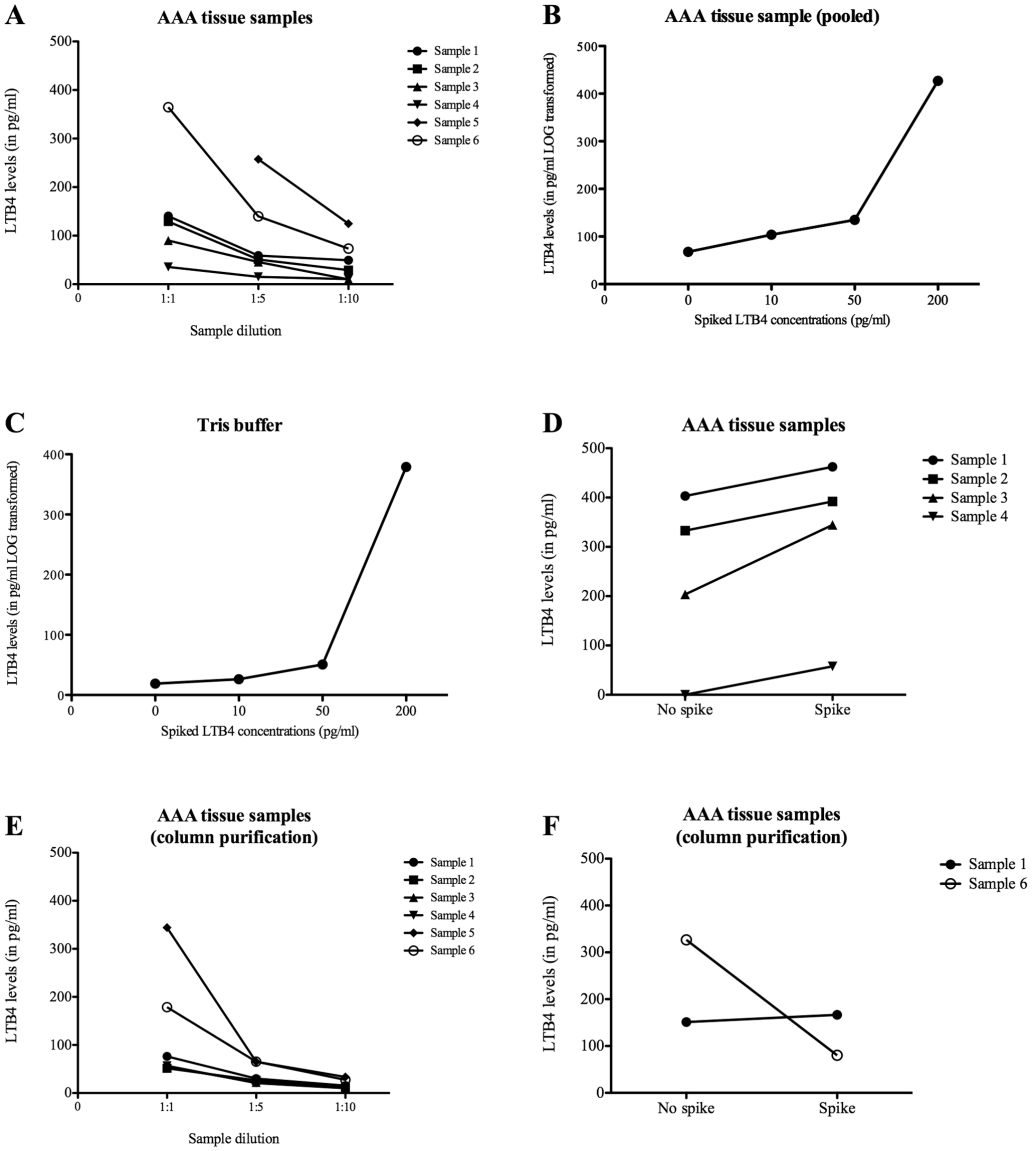
Validation of LTB4 measurements using ELISA. For validation of LTB4 measurements in tissue homogenates, six randomly chosen AAA tissue samples were used for subset analysis using LTB4 spiking and sample purification using lipid extraction (SPE (C-18) column). LTB4 levels were measured in six randomly chosen AAA samples in different dilutions (A). LTB4 spike recovery was performed in a pooled AAA sample (B) and Tris buffer only (control) (C) using different LTB4 concentrations. LTB4 spike recovery was also performed in individual AAA samples and compared to no spiking (D). Sample purification was performed on the same six randomly chosen AAA samples using the SPE (C-18) column purification method (E). LTB4 spike recovery was analyzed by adding a LTB4 spike (50 pg/ml) to the samples prior to column purification (n = 2) (F). LTB4 levels are expressed in pg/ml.

A pilot study was performed on a set of AAA tissue homogenates for validation of LTB4 measurements using ELISA after protein isolation. Lipid extractions were performed according to the SPE (C-18) column purification protocol described in the LTB4 EIA protocol by Cayman Chemicals (LTB4 EIA kit, Cayman Chemicals LtD, Michigan, USA). This protocol is designed for lipid extraction for lipid analysis. Non-purified samples were used for comparison. Dilution series were performed on non-purified and purified samples for accuracy of LTB4 measurements. In addition, column purifications were combined with LTB4 spiking (50 pg/ml) to validate accuracy of LTB4 measurements. All results are described in figure 1.

#### Protein isolation from CEA specimens

Adjacent segments of the culprit lesion were used for protein isolation as described previously [14]. In short, plaque segments were snap-frozen in liquid nitrogen and stored at −80°C until further use. Protein extraction was performed according to a standardized protocol using Tris buffer. Total protein concentration was determined using the BCA Protein Assay Kit (Thermo Fisher Scientific, Etten-Leur, The Netherlands). Expression levels of LTB4 within the atherosclerotic specimen were quantified using ELISA (LTB4 Parameter Assay Kit, R&D Systems Inc., Minneapolis, USA). LTB4 levels were adjusted for total protein concentration within the atherosclerotic specimen and expressed as pg/mg of total protein. For validation purposes, uncorrected LTB4 data is also depicted and expressed as pg/ul sample. Expression of a selection of proteins involved upstream and downstream of the ALOX5 pathway was analyzed using Western Blot and quantified as intensity per square millimeter. These were BLT1, ALOX5 and PKC.

#### Protein isolation from AAA specimens

Adjacent segments of AAA specimens were used for protein isolation as described previously [15]. In short, AAA segments were snap-frozen in liquid nitrogen and stored at −80°C until further use. Protein extraction was performed according to a standardized protocol using Tris buffer. Total protein concentration was determined using the BCA Protein Assay Kit (Thermo Fisher Scientific, Etten-Leur, The Netherlands). Expression levels of LTB4 within the AAA specimen were quantified using ELISA (LTB4 EIA kit, Cayman Chemicals LtD, Michigan, USA). LTB4 levels were adjusted for total protein concentration within the AAA specimen and expressed as pg/mg of total protein. For validation purposes, uncorrected LTB4 data is also depicted and expressed as pg/ul sample.

### Plasma LTB4 Levels (CEA)

At the time of inclusion, blood was collected in Sodium Citrate vacutainers at the outpatient clinic prior to surgery from a subpopulation of 35 randomly selected CEA patients. Blood was immediately centrifuged at 1850 G and subsequently isolated for LTB4 plasma measurements. To assess the comparison between plaque and plasma levels, LTB4 plasma levels were quantified using ELISA (LTB4 Parameter Assay Kit, R&D Systems Inc., Minneapolis, USA) and expressed as pg/ml of plasma. To validate the occurrence of *ex vivo* LTB4 formation after whole blood collection, a pilot validation study was performed with EDTA plasma (n = 4) and Sodium Citrate plasma (n = 4) preincubated with a 5-LOX inhibitor (100 µM Zileuton, Sigma Aldrich) or PBS (control) immediately after collection for 30 minutes. Plasma was collected and LTB4 levels were measured using ELISA (LTB4 Parameter Assay Kit, R&D Systems Inc., Minneapolis, USA) and expressed as pg/ml of plasma.

### Control Groups

Seven non-atherosclerotic mammary artery specimens were collected from patients scheduled for coronary artery bypass grafting (CABG). In addition, twenty-six non-aneurysmatic aortic specimens were collected from the infrarenal aortic wall by a pathologist during autopsy. Abdominal aortas exhibiting aneurysms or rupture were excluded. Specimens were processed identically to the CEA and AAA specimens as described above.

### Follow-up and Outcome

Primary outcome for both studies was defined as any vascular event or vascular intervention. This composite endpoint included non-fatal stroke, non-fatal myocardial infarction or perivascular intervention that was not planned at the time of inclusion (e.g. carotid surgery or angioplasty, coronary bypass, percutaneous coronary intervention, peripheral vascular surgery or angioplasty). Perivascular intervention comprised leg amputation and peripheral arterial intervention during follow-up that had not been planned at the time of inclusion [14], [15].

For this purpose, each patient received a yearly questionnaire informing if they experienced any vascular event or had been hospitalized in the past year and in the three years following surgery. If any of the questions were answered positively, further research was conducted to define outcome events. Following a standard scheme, discharge letters, and if needed, laboratory measurements and results of additional studies such as electrocardiograms or imaging studies were collected from the institution where the potential event occurred. If any patient did not respond to the follow-up, questionnaire, the general practitioner was contacted. Per potential event, two independent members of the outcome committee validated the information available. If the two members disagreed, a third outcome option was requested [14], [15].

### Statistical Analysis

SPSS (version 20.0, SPSS Inc., USA) was used for all statistical analyses. To determine sample size, a power calculation was performed for number of events versus controls (secondary manifestation during follow-up versus no secondary manifestation) for both the CEA and AAA samples (power of 80%, alpha of 0.05). Samples were randomly selected from both biobank databases. Data were expressed as mean ± standard deviation (SD) or median with interquartile range [IQR]. Correlations between different parameters were analyzed using Pearson’s Bivariate or a Spearman’s Bivariate correlation test. Statistical differences were analyzed using Student’s T test or one-way ANOVA (parametric data) or Mann Whitney U test or Kruskal-Wallis test (non-parametric data). Differences with p-values <0.05 were regarded as being significant.

## Results

### Validation of LTB4 Measurements Using ELISA

For validation of LTB4 measurements in tissue homogenates, six randomly chosen AAA tissue samples were used for subset analysis using lipid extraction (SPE (C-18) column purification) and LTB4 spiking. In a set of six non-purified samples LTB4 levels were measured in three different dilutions (1∶1, 1∶5 and 1∶10), diluted in EIA buffer. All measured LTB4 levels were consistent according to its dilution and fell in the linear range of the standard curve (except for sample 5 1:1) (figure 1a). Next, a pooled sample and Tris buffer alone were spiked using a series of LTB4 to measure spike recovery (0, 50, 100 and 200 pg/ml LTB4). As seen in figure 1b and 1c, respectively, the spiked concentrations were consistently reflected in the LTB4 levels measured (LOG transformed). In addition, a set of 4 AAA samples were used for LTB4 spiking (50 pg/ml). LTB4 spike recovery was traced back in all samples (figure 1d).

Next, sample purification was performed on the identical six randomly chosen AAA samples using the SPE (C-18) column purification method. As compared to figure 1a, observed LTB4 levels after column purification were lower in most samples and did not accurately reflect the dilutions (figure 1e). Lastly, LTB4 spike recovery was analyzed by adding a LTB4 spike (50 pg/ml) to the samples prior to column purification (n = 2). As shown in figure 1f, LTB4 spike recovery could not be traced back after column purification.

LTB4 measurements in both CEA and AAA samples were performed in Tris isolated tissue samples without prior lipid extraction (SPE (C-18) column purification).

### Histology of ALOX5 Pathway

**Figure 2:**
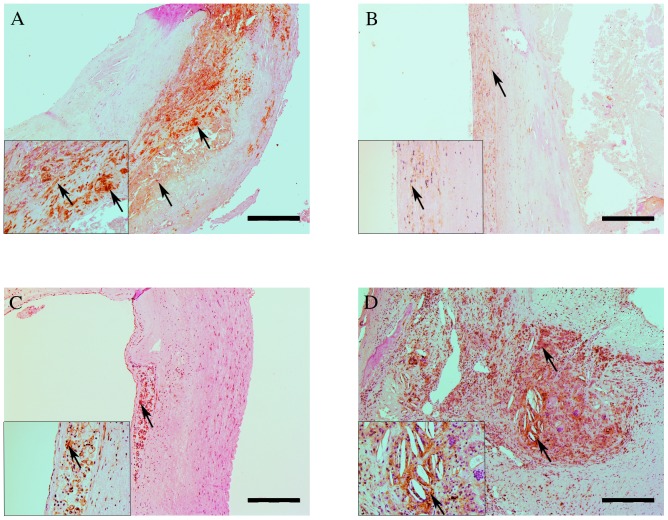
Immunohistochemical staining of BLT1 and ALOX5 in human atherosclerotic plaques. BLT1 expression could mainly be observed in areas rich in (foamy) macrophages (A), in both a membranous and cytoplasmatic expression. A weaker expression of BLT1 could be observed in areas rich of SMCs (B). ALOX5 expression could predominantly be observed in areas rich in (foamy) macrophages (C), and around cholesterol clefts (D). Arrows indicate positive staining (brown color). Scale bar: 500 µm. The insert shows 5x extra magnification.

BLT1 staining was broadly observed in different regions of the atherosclerotic plaque. Predominantly, the staining could be observed in regions with (foamy) macrophages. Both a membranous and a cytoplasmic staining were observed. In regions with SMC, only a weak BLT1 staining was present (figure 2a and b). In some cases, neutrophils in the thrombus of the plaque stained positive for BLT1. ALOX5 staining can be observed in the same areas as BLT1, mostly in areas with (foamy) macrophages and around cholesterol clefts (figure 2c and d). Arrows indicate positive staining (brown color).

### LTB4 Levels in Non-diseased Arterial Tissue

For comparison purposes, seven mammary artery specimens and twenty-six normal aortic specimens were used for LTB4 measurements.

LTB4 levels in mammary artery tissue were significantly lower compared to carotid atherosclerotic plaque levels (mammary artery tissue vs plaque; mean ± SD; 121±26 pg/mg vs 151±96 pg/mg; p = 0.025).

LTB4 levels in normal abdominal aortic tissue were also significantly lower compared to AAA levels (normal aortic tissue vs AAA; median [IQR]; 92 [27–381] pg/mg vs 246 [115–430] pg/mg; p = 0.017).

### LTB4 Levels in CEA and Patient Characteristics

Baseline patient characteristics of the 375 included CEA patients are provided in table 1. LTB4 levels in plaque tissue did not differ for gender, current smoking, diabetic status, hypertension treatment or Body Mass Index (BMI). However, CEA patients who suffered from hypercholesterolemia had significantly higher LTB4 levels (p = 0.037; see table 1). Furthermore, clinical presentation (asymptomatic versus symptomatic) or medication use did not influence LTB4 levels in CEA tissue. LTB4 levels were slightly higher in asymptomatic patients, however this difference did not reach statistical significance (p = 0.408).

**Table 1:** Clinical characteristics of patients undergoing carotid endarterectomy in relation with LTB4 expression.

Patient characteristics		LTB4 conc. (pg/mg protein) mean ± SD	P-value
Age (years) (mean **±** SD) (range)	67**±**9 (66–68)		
*Gender*			
Male	74.1%	145±83	
Female	25.9%	153±98	0.480
*Current smoker*			
No	62.4%	153±97	
Yes	37.6%	147±91	0.581
*Diabetic status*			
No	78.9%	150±95	
Yes	21.1%	153±94	0.803
*Hypertension*			
No	11.7%	158±85	
Yes	88.3%.	150±96	0.594
*Hypercholesterolemia*			
No	36.8%	137±96	
Yes	63.2%	159±93	0.037*
*Body Mass Index*			
BMI <25	34.4%	149±86	
BMI >25	65.6%	155±100	0.582
*History peripheral intervention*			
No	79.6%	151±94	
Yes	20.4%	153±96	0.861
*History myocardial infarction*			
No	79.5%	156±94	
Yes	20.5%	133±94	0.055
*Statin use*			
No	25.3%	140±91	
Yes	74.7%	155±95	0.207
*Aspirin use*			
No	14.2%	145±97	
Yes	85.8%	151±94	0.647
*Oral anti-coagulant use*			
No	90.1%	154±92	
Yes	9.9%%	129±103	0.063
*ACE inhibitor use*			
No	66.4%	155±94	
Yes	33.6%	141±95	0.199
*Angiotensin-II antagonist use*			
No	79.5%	149±92	
Yes	20.5%	156±105	0.533
*Serum levels*			
C-reactive protein (CRP) (mean ± SD)	7.23±17.165		
HDL (mmol/L) (mean ± SD)	1.149±0.374		
LDL (mmol/L) (mean ± SD)	2.814±1.048		
*Clinical presentation*			
Asymptomatic	15.7%	154±84	
Amaurosis Fugax	11.7%	152±94	
TIA	41.6%	148±91	
(Minor) Stroke	23.7%	149±95	
*Asymptomatic vs symptomatic*			
Asymptomatic	15.7%	154±84	
Symptomatic (TIA & (minor) Stroke)	65.3%	148±93	0.408

LTB4 plaque expression levels are expressed as mean ± standard deviation.

Abbreviations: ACE: Angiotensin Converting Enzyme, HDL: high-density lipoprotein, LDL: low-density lipoprotein, LTB4: Leukotriene B4, SD: standard deviation. Patient characteristics have been correlated with continuous LTB4 expression plaque levels. P-values were calculated using Student’s T test. Differences with p-value <0.05 was regarded significant marked an with asterisk (*).

### LTB4 Levels in AAA and Patient Characteristics

Baseline patient characteristics of the 241 included AAA patients are provided in table 2. LTB4 levels in AAA tissue did not differ for gender, current smoking, diabetic status, hypertension treatment or BMI. However, AAA patients who suffered from hypercholesterolemia had significantly higher LTB4 levels (p = 0.046; see table 2). Furthermore, clinical presentation or medication use did not influence LTB4 levels in AAA tissue. LTB4 levels were slightly higher in elective patients, however this difference did not reach statistical significance (p = 0.108).

**Table 2:** Clinical characteristics of patients undergoing open AAA repair in relation with LTB4 expression.

Patient characteristics		LTB4 conc. (pg/mg protein) median [IQR]	P-value
Age (years) (mean **±** SD) (range)	70±7 (69–71)		
*Gender*			
Male	83.8%	254 [117–428]	
Female	16.2%	166 [98–494]	0.305
*Current smoker*			
No	51.8%	255 [117–463]	
Yes	48.2%	246 [125–419]	0.486
*Diabetic status*			
No	88.6%	256 [115–416]	
Yes	11.4%	220 [113–520]	0.925
*Hypertension*			
No	29.7%	200 [103–349]	
Yes	70.3%	268 [104–511]	0.092
*Hypercholesterolemia*			
No	38.6%	194 [113–360]	
Yes	61.4%	280 [125–526]	0.046*
*Body Mass Index*			
BMI <25	38.5%	228 [127–378]	
BMI >25	61.5%	337 [128–518]	0.062
*History myocardial infarction*			
No	69.9%	230 [118–434]	
Yes	30.1%	259 [114–451]	0.780
*Statin use*			
No	35.7%	197 [96–357]	
Yes	64.3%	276 [124–522]	0.057
*Aspirin use*			
No	79.8%	225 [110–434]	
Yes	21.2%	303 [110–434]	0.284
*Oral anti-coagulant use*			
No	84.8%	267 [114–442]	
Yes	15.2%	224 [123–399]	0.968
*ACE inhibitor use*			
No	67.9%	227 [126–503]	
Yes	32.1%	195 [100–391]	0.410
*Angiotensin-II antagonist use*			
No	77.4%	249 [118–414]	
Yes	22.6%	271 [79–518]	0.983
*Serum levels*			
C-reactive protein (CRP) (mean ± SD)	15.32±37.97	(7.94–22.71)	
HDL (mmol/L) (mean ± SD)	0.980±0.350	(0.91–1.04)	
LDL (mmol/L) (mean ± SD)	2.770±2.56	(2.56–2.97)	
*Clinical presentation*			
Elective	78.4%	267 [129–466]	
Symptomatic	12.0%	191 [48–331]	
Ruptured	9.6%	193 [67–559]	0.108
*Asymptomatic vs symptomtic*			
Asymptomatic (elective)	78.4%	267 [129–466]	
Symptomatic (symptomatic & ruptured)	21.6%	192 [67–349]	0.049*

LTB4 abdominal aortic aneurysm expression levels are expressed as median [IQR].

Abbreviations: AAA: Abdominal Aortic Aneurysm, ACE: Angiotensin Converting Enzyme, HDL: high-density lipoprotein, LDL: low-density lipoprotein, LTB4: Leukotriene B4, SD: standard deviation. Patient characteristics have been correlated with continuous LTB4 expression aneurysm levels. P-values were calculated using Kruskal-Wallis or Mann-Whitney U test. Differences with p-value <0.05 was regarded significant marked with an asterisk (*).

### LTB4 Levels in CEA and Tissue Characteristics

**Figure 3:**
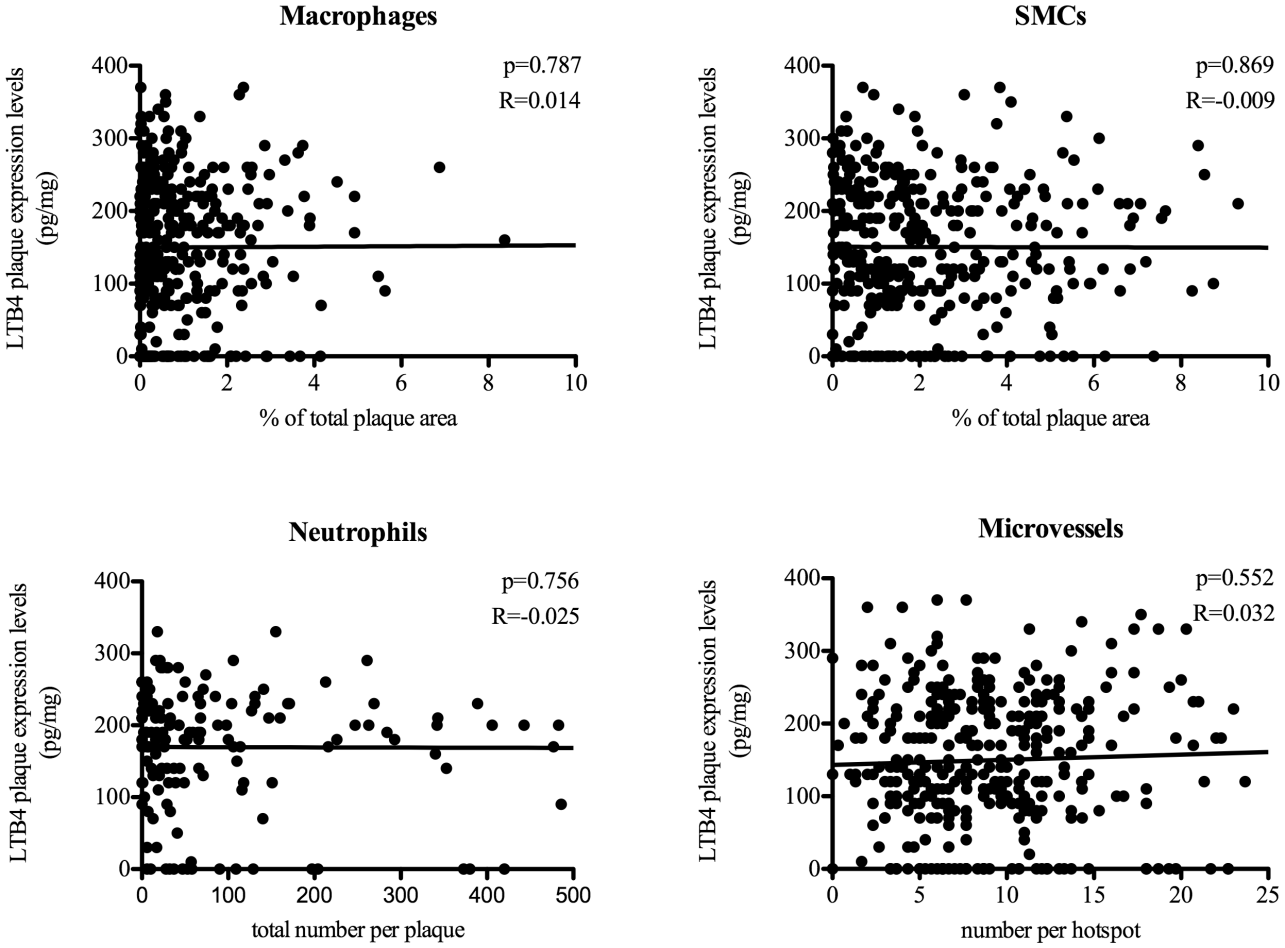
Correlation between LTB4 plaque expression levels and quantitative analysis of different cell components. Dot plots of macrophage, smooth muscle cell, neutrophil and microvessel content, scored using computerized analysis in relation to LTB4 plaque expression levels. Macrophage and smooth muscle cell content were expressed as a percentage of total plaque area. Neutrophil content was expressed as total number of neutrophils of total plaque area. Microvessel numbers were scored and expressed per hotspot. Correlations were analyzed using Pearson’s Bivariate correlation test. Differences with a p-value <0.05 were regarded as being significant. LTB4 levels are expressed in pg/mg.

**Figure 4:**
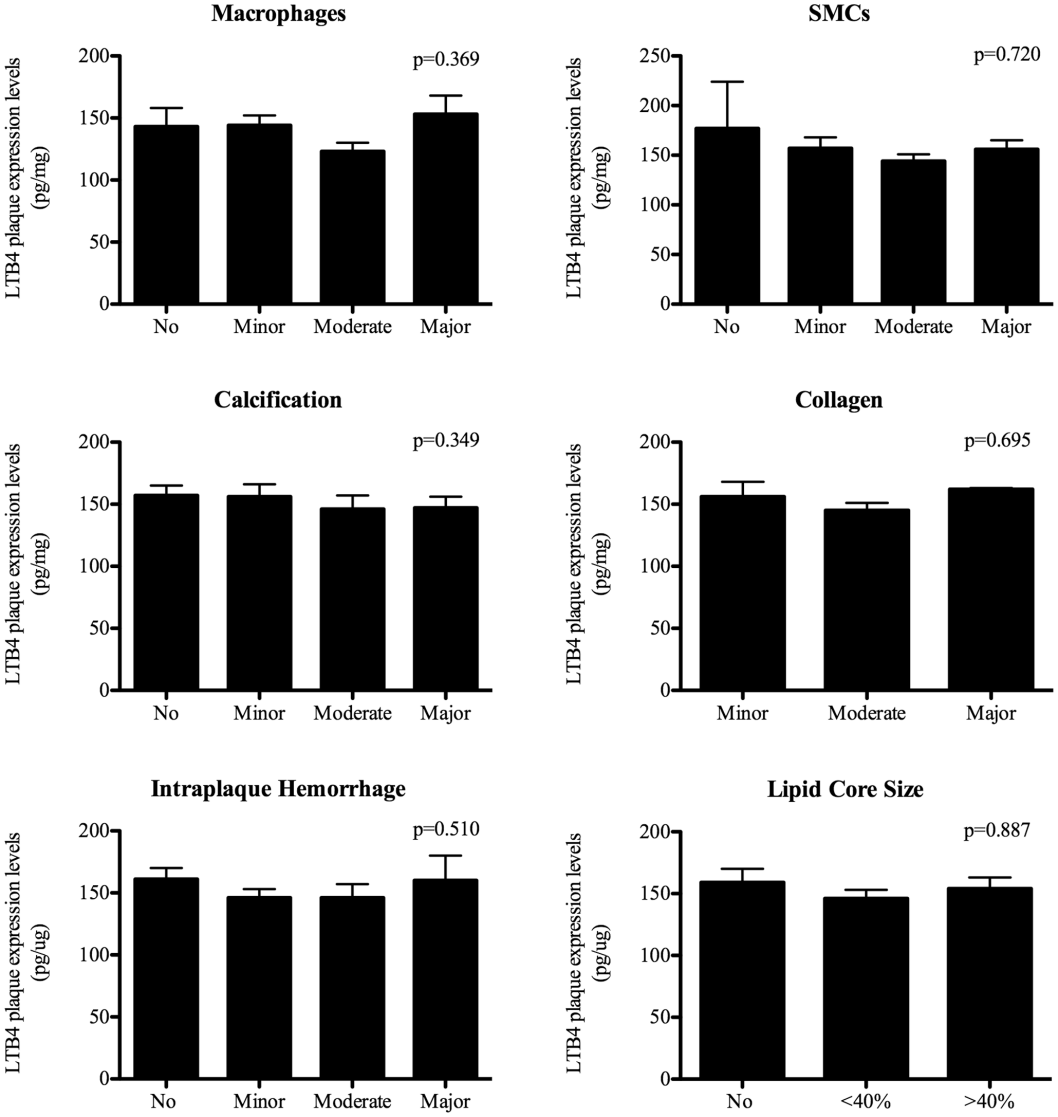
Comparison of LTB4 plaque expression levels and histological plaque composition of atherosclerotic carotid plaques. Bar graphs of LTB4 plaque expression levels in relation with semi-quantative histological plaque characteristics. All characteristics have been correlated with continuous LTB4 expression levels and analyzed by Pearson Bivariate correlation test. Differences with p-value <0.05 were regarded as being significant. All data is expressed as mean ± standard deviation. LTB4 levels are expressed in pg/mg. Abbreviations: SMC: smooth muscle cell content.

**Figure 5:**
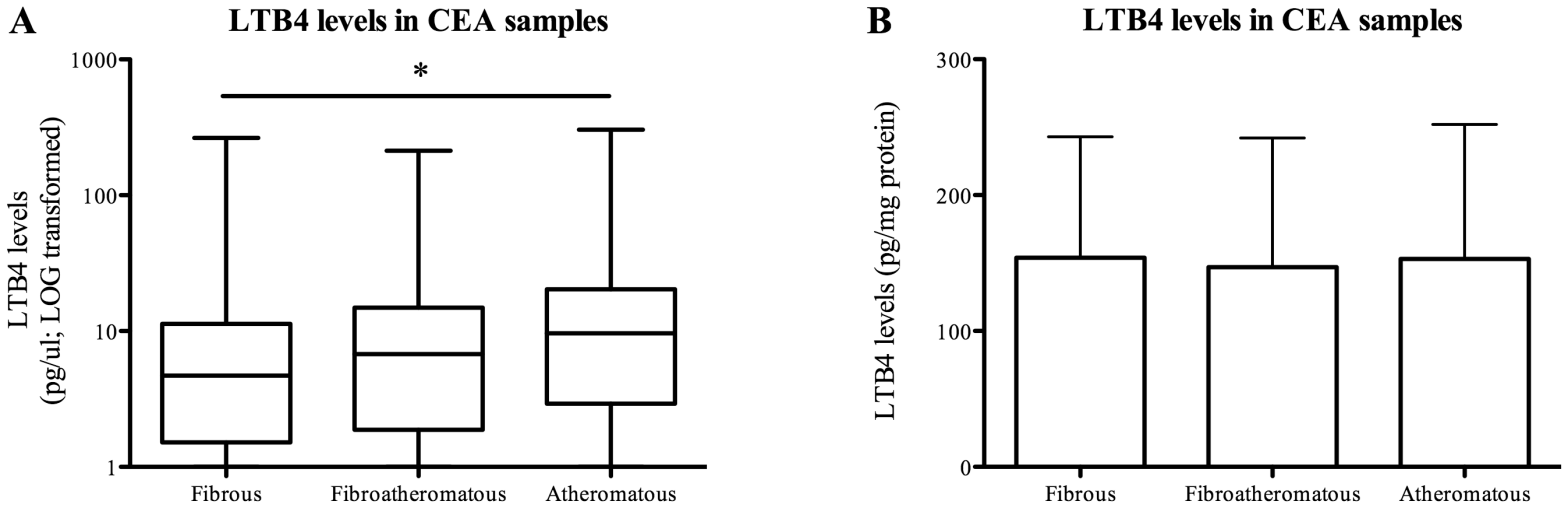
Uncorrected versus corrected LTB4 levels and plaque phenotype. LTB4 levels in atherosclerotic plaques were measured using ELISA and corrected for total protein content. Uncorrected and corrected levels were related to the three plaque phenotypes: fibrous (n = 113), fibroatheromatous (n = 131) and atheromatous (n = 130). Uncorrected LTB4 levels were positively related with an atheromatous plaque phenotype (A, p = 0.005). After correction, this association was not present (B, p = 0.791). Data is presented as median with interquartile range [IQR] and in pg/ul for uncorrected data and mean ± standard deviation and in pg/mg for corrected data.

Histological examination of atherosclerotic plaques revealed no correlations between LTB4 levels and the quantitatively measured macrophage, SMC or neutrophil numbers (figure 3). The additional semi-quantitative analyses of macrophage and SMC content supported these findings (figure 4). No correlations were observed between LTB4 levels and other plaque characteristics defining plaque phenotype, such as plaque calcifications, collagen content, lipid core size and intraplaque hemorrhage (figure 4). Plaque microvessel density quantified per hotspot was also not correlated to LTB4 levels (figure 3). Proteins involved in the ALOX5 pathway were quantified using Western Blot in atherosclerotic plaques. Expression of these proteins, BLT1, PCK and ALOX5, did not significantly correlate with inflammatory cell numbers in the plaque (data not shown). Uncorrected LTB4 levels were positively associated with an atheromatous plaque phenotype (p = 0.005), whereas after protein correction this association was not present (p = 0.791) (figure 5).

### LTB4 Levels in AAA and Tissue Characteristics

**Figure 6:**
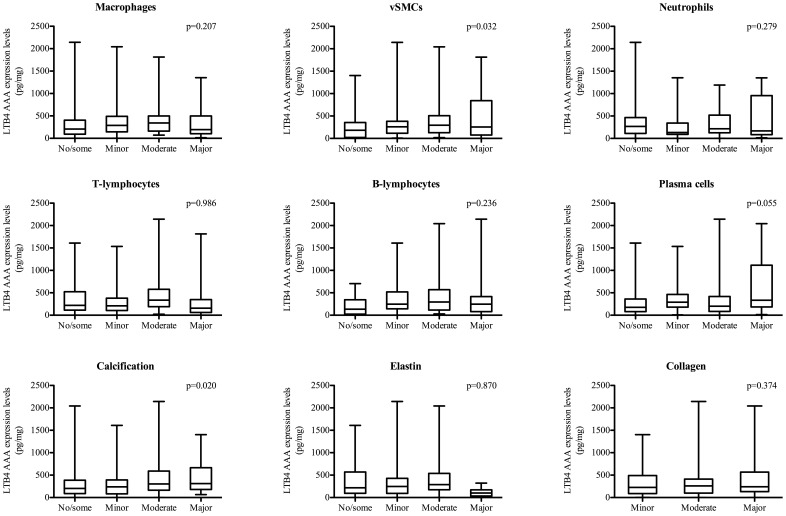
Comparision of LTB4 Abdominal Aotic Aneurysm (AAA) expression levels and histological tissue composition of AAA. Box-plots of LTB4 abdominal aortic aneurysm expression levels in relation with semi-quantative histological AAA characteristics. All characteristics have been correlated with continuous LTB4 expression levels and analyzed by Spearman’s Bivariate correlation test. Differences with p-value <0.05 were regarded as being significant. All data is expressed as median with interquartile range [IQR] and in pg/mg. Abbreviations: SMC: smooth muscle cell content.

Histological analysis of AAA specimens only revealed a significant positive correlation between LTB4 levels and the presence of vascular SMCs (p = 0.032) and the presence of calcifications (p = 0.021). No other significant correlations were observed with other histological markers in AAA (figure 6).

### LTB4 Levels in CEA and Clinical Follow-up

**Figure 7:**
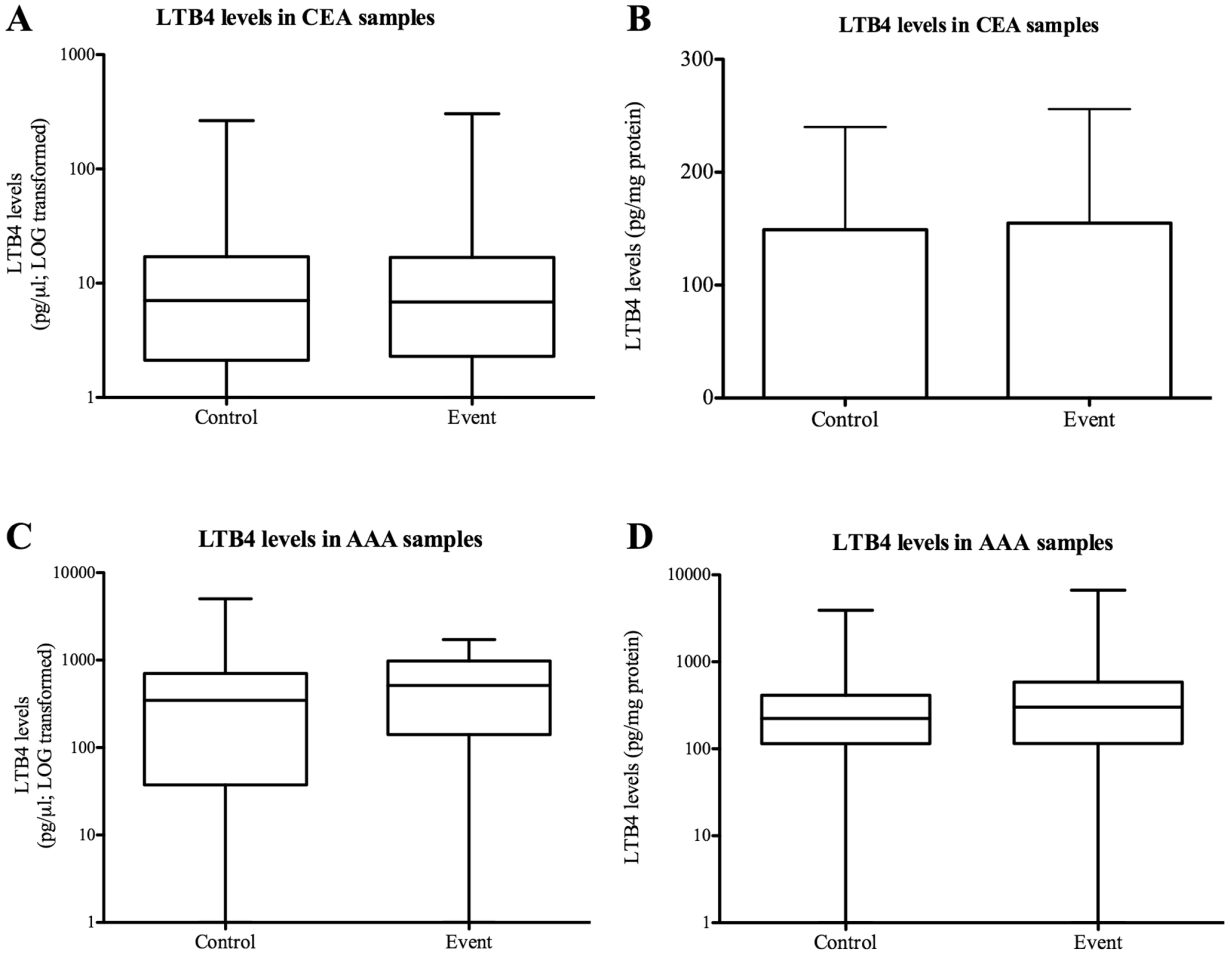
Uncorrected versus corrected LTB4 levels and secondary clinical outcome. LTB4 levels in atherosclerotic plaques and AAA tissue were measured using ELISA and corrected for total protein content. For validation purposes, also uncorrected data are depicted in these graphs. Uncorrected and corrected LTB4 levels were related to control versus events (no secondary outcome versus secondary outcome during follow-up). Both corrected and uncorrected LTB4 levels were not significantly different between controls and events for both CEA (A and B) and AAA (C and D) patients. Data is presented as mean ± standard deviation or median with interquartile range [IQR] and in pg/ul for uncorrected data and in pg/mg for corrected data.

The Athero-Express study previously demonstrated that specific plaque proteins or histological characteristics measured in local atherosclerotic plaques can be predictive for events that originate from all vascular territories [16], [18]. In the AE database, the mean follow-up time was 2.7 years (SD 0.86, maximum = 5.74). During this period, 142 patients (37.9%) reached a clinical endpoint (table 3). During follow-up, 38 patients suffered from a non-fatal stroke (10.1%), 46 patients suffered from non-fatal myocardial infarction (12.3%), and 69 patients suffered from peripheral vascular intervention (18.4%). No differences were found in LTB4 levels between patients who reached a clinical endpoint or not (event vs control; mean ± SD; 151±101 pg/mg vs. 149±91 pg/mg; p = 0.549). In table 3, all primary clinical endpoints are described separately. Uncorrected LTB4 levels were also not associated with secondary clinical outcome, similar to the corrected LTB4 levels (figure 7a and b).

**Table 3:** Characteristics with respect to follow-up and LTB4 tissue levels in CEA patients.

Baseline characteristics		
Mean follow-up (years) (SD, maximum)	2.7	(0.86, 5.74)
Primary outcome (3 years FU)	142/375	37.9%
**Primary clinical outcome**		
Non-fatal stroke (n; %)	38/375	10.1%
Non-fatal myocardial infarction (n; %)	46/375	12.3%
Peripheral vascular intervention (n; %)	69/375	18.4%
**Primary clinical outcome and LTB4 levels**		**P-value**
Control (mean ± SD)	149±91	
Event (mean ± SD)	155±101	0.549

LTB4 plaque expression levels are expressed as mean ± standard deviation and as pg/mg protein. P-values were calculated using Student’s T test. Differences with p-value <0.05 was regarded as being significant.

Abbreviations: CEA: carotid endarterectomy, FU: follow-up, LTB4: leukotriene B4, SD: standard deviation.

### LTB4 Levels in AAA and Clinical Follow-up

For the AAA patients, the mean follow-up time was 2.0 years (SD 1.17, maximum = 4.89). During this period, 36 patients (14.5%) reached a clinical endpoint (table 4). During follow-up, 12 patients died (5.0%), 9 patients suffered from a non–fatal stroke (3.7%), 14 patients suffered from non-fatal myocardial infarction (5.8%) and 1 patient suffered from peripheral vascular intervention (0.4%). No significant differences were found in LTB4 levels between patients reaching a clinical endpoint or not (control vs event; median [IQR]; 224 [115–414] pg/mg vs 302 [115–584] pg/mg; p = 0.215). In table 4, all primary clinical endpoints are described separately. Uncorrected LTB4 levels were also not associated with secondary clinical outcome, similar to the corrected LTB4 levels (figure 7c and d).

**Table 4:** Characteristics with respect to follow-up and LTB4 tissue levels in AAA patients.

Baseline characteristics		
Mean follow-up (years) (SD, maximum)	2.0	(1.17; 4.89)
Primary outcome (3 years FU)	36/241	14.5%
**Primary clinical outcome**		
Cardiovascular death (n; %)	12/241	5.0%
Non-fatal stroke (n; %)	9/241	3.7%
Non-fatal myocardial infarction (n; %)	14/241	5.8%
Peripheral vascular intervention (n; %)	1/241	0.4%
**Primary clinical outcome and LTB4 levels**		**P-value**
Control (median [IQR])	224 [115–414]	
Event (median [IQR])	302 [115–584]	0.215

LTB4 abdominal aortic aneurysm expression levels are expressed as median [IQR] and as pg/mg protein. P-values were calculated using Mann-Whitney U test. Differences with p-value <0.05 was regarded as being significant.

Abbreviations: AAA: Abdominal Aortic Aneurysm, FU: follow-up, IQR: interquartile range, LTB4: leukotriene B4 SD: standard deviation.

### LTB4 Plasma Levels in CEA and Patient Characteristics and Clinical Follow-up

**Figure 8:**
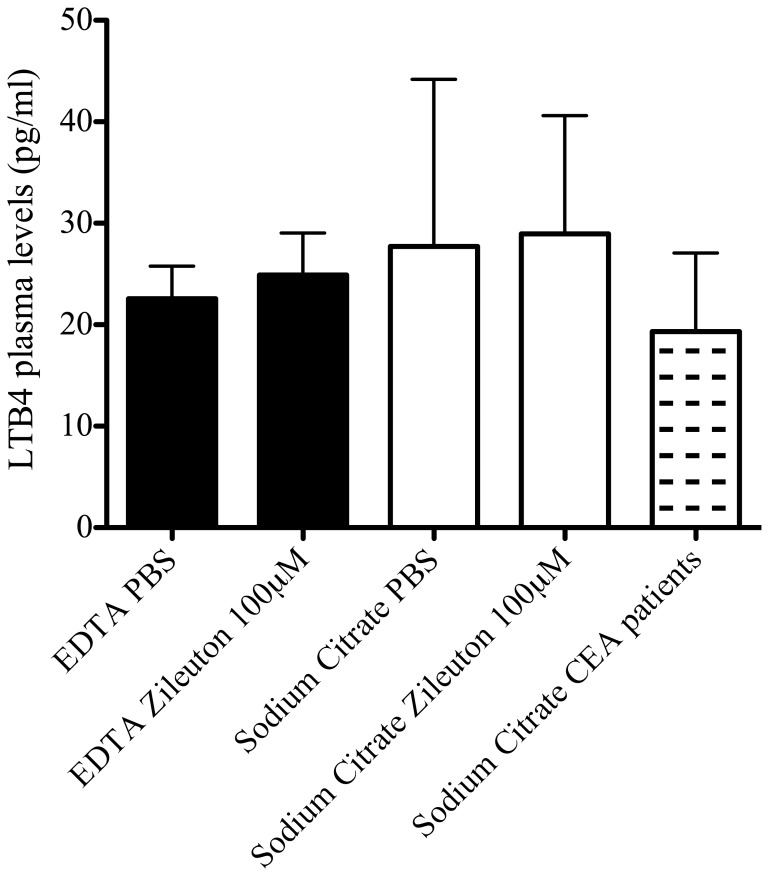
Validation of LTB4 measurements in plasma using ELISA. Validation of *ex vivo* LTB4 production after whole blood collection by EDTA (black bars, n = 4, duplo) or Sodium Citrate (white bars, n = 4, duplo) Vacutainers. Directly after collection, whole blood was incubated with a specific 5-LOX inhibitor Zileuton (100 µM for 30 minutes) and plasma was stored. LTB4 was measured using ELISA and compared with levels of CEA patients (n = 35). Data is expressed as mean ± standard deviation and in pg/ml.

LTB4 plasma levels were measured in 35 randomly selected CEA blood samples (9.3%) from the AE database. LTB4 plasma levels did not correlate with plaque levels (R-0.093, p = 0.60). No significant correlations were found between LTB4 plasma levels and baseline patient characteristics or clinical outcome after correcting for multiple testing. Validation experiments excluded *ex vivo* LTB4 formation after whole blood collection (figure 8).

### LTB4 Plasma Levels in CEA and Tissue Characteristics

Histological examination of atherosclerotic plaques revealed no correlations between LTB4 plasma levels and the quantitative macrophage, SMC or neutrophil analyses. The additional semi-quantitative analyses of macrophage and SMC content supported these findings. No correlations were observed between LTB4 plasma levels and other plaque characteristics defining plaque phenotype, such as plaque calcifications, collagen content, lipid core size and intraplaque hemorrhage. Plaque microvessel density quantified per hotspot was also not correlated to LTB4 plasma levels (data not shown).

## Discussion

In the present study, we performed a parallel analysis of LTB4 levels in two large independent databases consisting of either human advanced atherosclerotic plaques or human AAA tissue and studied its relation to tissue characteristics and clinical outcome. We hypothesized that LTB4 levels were higher in vulnerable atherosclerotic plaques. Furthermore, we hypothesized that LTB4 levels were higher in inflammatory AAA and that it was related to adverse clinical outcome. We found that LTB4 levels were measurable in both human atherosclerotic plaques and AAA tissue.

LTB4 is a potent chemoattractant involved in inflammatory diseases and is known to play a major role in the initiation and progression of atherosclerosis. Animal studies have shown that combining ApoE or LDLR deficiency with BLT1 deficiency or antagonizing the BLT1 receptor all results in a marked decrease of the formation of atherosclerotic lesions [19]–[21]. In vitro, LTB4 induces monocyte chemoattractant protein (MCP) −1 production in isolated human monocytes [22]. Furthermore, LTB4 can act on vascular SMCs by activation, migration and proliferation via the BLT1 receptors [23]. LTB4 may not only play a role in the initiation and progression of atherosclerosis, but also in the destabilization of atherosclerotic plaques over time. In human atherosclerotic plaques, constituents of the ALOX5 pathway are expressed at high levels in patients suffering from a stroke, compared to asymptomatic patients [24]. The expression of ALOX5 and its receptor BLT1 have also been described across the human AAA specimens as well. In both murine and human AAAs, expression of ALOX5 and BLT1 is found by macrophages, neutrophils and T-lymphocytes [25], [26]. These data show that different components of the ALOX5 pathway are involved in the formation and progression of both atherosclerosis and AAA.

LTB4 may also be associated with plaque rupture in advanced human atherosclerotic plaques. A link has been suggested between LTB4 production and increased matrix metalloproteinase levels and its activity in both plaques [23], [27] and AAA [25], [26]. In previous studies, elevated levels of different components of the ALOX5 pathway have been related to plaque and AAA instability. Consequently, this phenomenon has led to the development of antagonists for different components of the ALOX5 pathway [28] for instance the BLT1 receptor [29]. However, clinical trials have not yet confirmed any effects on atherosclerosis or AAA development, mainly because consistent evidence on the role of LTB4 in advanced atherosclerosis and AAA in humans is lacking.

In our study, we found no association between LTB4 levels in atherosclerotic plaques and markers for inflammatory plaque phenotype, such as macrophage, lipid, SMC or collagen content. As a consequence, LTB4 levels were not associated with a more atheromatous (vulnerable) plaque phenotype. However, LTB4 levels without correction for protein content were positively associated with this phenotype. Therefore, the association of LTB4 levels with tissue characteristics of atherosclerotic plaques must be carefully considered. Next, we decided to investigate the expression of markers involved in the ALOX5 pathway in the plaques using immunohistochemistry. Previously, Cippolone *et al.* [24] observed a wide expression of ALOX5 in human plaques, mostly expressed by (foamy) macrophages. We can confirm this in our selection of plaques, moreover we observed BLT1 staining in our plaques expressed by different cell types, such as (foamy) macrophages and SMCs.

Earlier findings show that ALOX5 levels are related to markers of plaque instability [27]. We measured levels of not only ALOX5, but also PKC and BLT1 in a selection of CEA patients using Western Blot. However, we could not find any associations between these expression levels and markers related to the inflammatory plaque phenotype. Furthermore, Cippolone *et al*. [24] and Zhou *et al*. [27] showed that levels of both ALOX5 and LTB4 were significantly higher in symptomatic patients compared to asymptomatic patients and were significantly higher in diabetic patients compared to non-diabetic patients in a small cohort of sixty atherosclerotic plaques. We measured LTB4 plaque levels in a larger sample size. However, we were not able to confirm these findings in our patient cohort, nor did we find any relation to other cardiovascular risk factors. A discrepancy between distributions of different patient groups can be an explanation for the fact that we are not able to reproduce these data, although our study numbers should be sufficiently powered. Our cohort only comprises 15.7% asymptomatic and 21.1% diabetic patients, compared to 50% in the study of Cippolone *et al*. or Zhou *et al*. for symptomatic and diabetic status, respectively. Another explanation could be that in the last five years patients are being operated with a significant shorter delay following the event. Differences in the delay between primary events and surgery may explain alterations in plaque characteristics and hence differences in observations among these studies [30].

In our AAA cohort, LTB4 levels have been related to the inflammatory status of the tissue. The first report on proteins of the ALOX5 pathway in human AAA specimens by Houard *et al*. [25] showed that components of this pathway are well distributed in the tissue. Furthermore, they showed significant relations between LTB4 levels and components related to neutrophil activity. Therefore, we related LTB4 levels to cell components of the AAA wall. We observed no significant associations between inflammatory markers and LTB4 after correcting for multiple testing. Also, no associations were found between LTB4 levels and baseline patient characteristics in our AAA patient cohort.

Atherosclerosis is a systemic disease. The Athero-Express study is a longitudinal study with the underlying hypothesis that local plaque characteristics hold information about the stability of plaques in the whole vascular system. In our analysis of the isolated carotid atherosclerotic plaque, we assumed that local biomarkers found in this plaque show similarities within the whole vascular system and can predict systemic clinical cardiovascular outcome [16], [18]. In line with this concept that we previously applied successfully, we assessed the predictive value of local LTB4 expression for future adverse cardiovascular events. We report that LTB4 plaque levels were not related to clinical outcome during follow-up either with or without correction for protein content. Based on this observation we can only infer that there was no association between local plaque LTB4 levels and clinical outcome. No assumptions can be made regarding the effects of LTB4 during local plaque progression or destabilization.

The study rationale of the Aneurysm-Express biobank is similar to that of the Athero-Express. In line with our findings in plaques, we could not observe any associations with LTB4 levels in AAA tissue and clinical outcome. Also, no associations were observed without correction for protein content. Again, no assumptions can be made regarding the effects of LTB4 during AAA progression.

Within a subgroup of CEA patients, we also measured LTB4 blood plasma levels. These levels were not significantly associated with patient and plaque characteristics. This is in line with our findings from LTB4 plaque levels. In the past, Sánchez-Gálan *et al*. [31] showed in a small cohort of patients and healthy controls that LTB4 plasma levels were significantly lower in healthy controls compared to atherosclerotic patients.

For comparison purposes, we decided to measure LTB4 levels in a small set of appropriate control tissues for both plaque and AAA tissues. We compared LTB4 levels of mammary arteries to the LTB4 levels of the atherosclerotic plaques and the LTB4 levels of aortic specimens to the LTB4 levels of the AAA tissue, independently. For both tissue types, we observed significantly lower levels in the control tissues compared to diseased tissues. As mentioned earlier, Sánchez-Gálan *et al*. [31] showed that LTB4 plasma levels were significantly lower in healthy subjects compared to atherosclerotic patients. In addition to this finding, we can now show that this also holds true for LTB4 levels in the vessel tissue not only in atherosclerosis, but also in AAA.

### Study Limitations

Our study comprises some limitations. As mentioned earlier, the study design of both the Athero-Express and Aneurysm-Express are cross-sectional studies and therefore we cannot make strong inferences regarding causality. The current study is not supportive for a causal role of tissue LTB4 in progression of human disease, but intervention studies are needed to provide a definite answer. Furthermore, in this study we mainly focused on LTB4 only and not all the components of the ALOX5 pathway that influence LTB4 production. We measured a selection of other components in this pathway in the atherosclerotic plaques, however this was done in a randomly selected subgroup within our patient group. In addition, expression levels of these other components were measured using Western Blot, a method not very suitable for quantification. Lastly, LTB4 plaque and AAA levels were measured without prior lipid extraction using column purification and levels were corrected for the total protein content. It merits careful consideration if these methods of measurement and expression are representative for LTB4, since this is known as a lipid mediator. However, we validated the performed methods in this study. We showed that lipid extraction using column purification resulted in potential loss of LTB4 when compared to non-purified samples. Taken this into account, we did not perform these lipid extractions. It should be emphasized that the conclusions drawn from the validation experiments are solely based on the results obtained in this study with the specific method and samples used. Therefore, these data cannot easily be extrapolated to other studies using different sample or tissue types. The measurements of LTB4 without prior lipid extractions are a limitation of this study. Still, these results were not encouraging and failed to show even a trend towards an association with tissue or clinical characteristics.

### Conclusions

From the current study, we may conclude that LTB4 levels within the advanced human carotid atherosclerotic plaque are not related to markers associated with plaque vulnerability. Furthermore, LTB4 plaque levels are not associated with baseline patient characteristics or adverse clinical outcome. In line with these findings, we could observe similar findings regarding LTB4 levels and AAA tissues. Therefore, LTB4 may play a role in the initiation and early progression of atherosclerotic disease and AAA in animal models; we could not provide supportive evidence for a detrimental role in advanced stages of human atherosclerosis or AAA.
